# Early recognition and management of Lemierre’s syndrome with extensive central venous thrombosis: a case from the emergency department

**DOI:** 10.1186/s12879-026-13376-6

**Published:** 2026-04-27

**Authors:** Mohammad K. Alsuleiman, Murad A. Yasawy

**Affiliations:** Department of Emergency Medicine, Faculty of Medicine, Jordan University of Science and Technology, Irbid, 22110 Jordan

**Keywords:** Lemierre’s syndrome, Jugular vein thrombophlebitis, Septic embolism

## Abstract

Lemierre’s syndrome is a rare but potentially life-threatening condition. It typically develops as a complication of oropharyngeal infections, leading to bacteremia, septic thrombophlebitis of the internal jugular vein (IJV), and metastatic septic emboli. According to the literature, Lemierre’s syndrome predominantly affects previously healthy young adults, with a mean age at presentation of around 20 years, although it has also been reported in school-aged children. Diagnosis is frequently delayed or missed, as the initial manifestations—such as sore throat or neck pain—closely resemble benign viral infections. Emergency physicians therefore play a critical role in recognizing early warning signs and initiating prompt, lifesaving management. This report describes an atypical presentation of Lemierre’s syndrome in a middle-aged (43-year-old) woman who exhibited minimal systemic toxicity despite extensive central venous involvement.

## Introduction

Lemierre’s syndrome, often referred to as the “forgotten disease,” is a rare but potentially life-threatening condition with an estimated global incidence of approximately 1 per 1,000,000 individuals. It typically develops as a complication of oropharyngeal infections, leading to bacteremia, septic thrombophlebitis of the internal jugular vein (IJV), and metastatic septic emboli. According to the literature, Lemierre’s syndrome predominantly affects previously healthy young adults, with a mean age at presentation of around 20 years, although it has also been reported in school-aged children. While *Fusobacterium necrophorum* remains the most common causative organism, an increasing number of culture-negative and atypical bacterial aetiologies have been described [1–4].

Diagnosis is frequently delayed or missed, as the initial manifestations—such as sore throat or neck pain—closely resemble benign viral infections. Emergency physicians therefore play a critical role in recognizing early warning signs and initiating prompt, lifesaving management. This report describes an atypical presentation of Lemierre’s syndrome in a middle-aged (43-year-old) woman who exhibited minimal systemic toxicity despite extensive central venous involvement.

### Ethical considerations

The Institutional Review Board (IRB) at the Faculty of Medicine, JUST, Jordan, revised and approved this case report under IRB No. (OCT2025/187 − 32). This case report was conducted in accordance with the principles outlined in the Declaration of Helsinki. Written informed consent was taken from the patient.

## Case presentation

A 43-year-old woman presented to our emergency department (ED) with a 4-day history of left-sided neck pain radiating to the left shoulder and chest, progressively worsening and unrelieved by over-the-counter analgesics. She reported mild fever earlier in the week and noted a brief episode of flu-like symptoms one week prior, which resolved spontaneously.

She had been evaluated at another hospital the day before, diagnosed with a “viral infection,” and discharged after receiving intravenous fluids and analgesics. The pain, however, intensified, prompting her emergency department visit.

She denied loss of consciousness, headache, visual changes, dysphagia, dyspnea, palpitations, abdominal pain, vomiting, urinary symptoms, or recent dental procedures. She had no history of chronic illnesses or surgeries and no known drug or food allergies. The patient was on progesterone-based oral contraceptive pills and smoked approximately three cigarettes per day.

### Physical examination

Vital signs: blood pressure (BP) 122/77 mmHg, heart rate (HR) 81 bpm, respiratory rate (RR) 16/min, Temperature (T) 36.7 °C, oxygen saturation (SpO₂) 97% on room air.

General: Alert, oriented, in visible pain but afebrile and hemodynamically stable.

Neck: Left-sided swelling, erythema, and tenderness extending from the submandibular region to the upper chest at the level of the first rib. No carotid bruit or fluctuance.

Throat: No erythema or exudates; airway patent.

Chest: Normal air entry bilaterally, no added sounds.

Neurological exam: Cranial nerves intact; motor and sensory exams normal except for limited left upper limb movement due to pain. No focal neurological deficit.

### Investigations

**Fig. 1:**
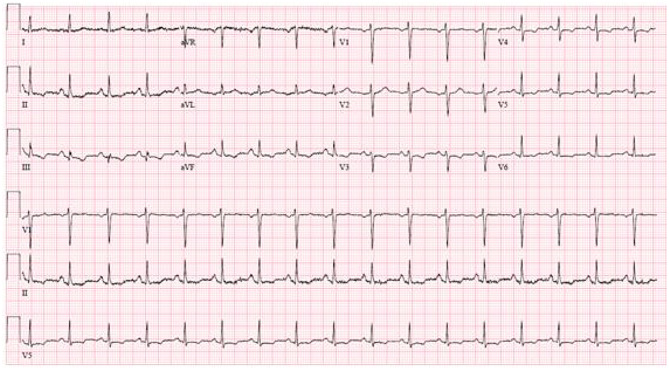
Electrocardiography (ECG) showing normal sinus rhythm, T-wave inversion in leads III, aVF, and V3–V6, which is similar to previous ECG of the same patient

Laboratory Findings (Table 1) and Electrocardiography (ECG) (Fig. 1) are detailed below:

**Table 1:** Laboratory Findings:

Test	Results	Reference Range
White blood cell (WBC)	14.0 × 10^3^/mm^3^	↑
Neutrophils	78.1%	↑
haemoglobin (Hb)	12.4 g/dL	Normal
Platelets	384 × 10^3^/mm^3^	Normal
Creatinine	57 µmol/L	Normal
Urea	3.3 mmol/L	Normal
Sodium (Na)	142 mmol/L	Normal
Potassium (K)	4.01 mmol/L	Normal
C reactive Protein (CRP)	170 mg/L	↑
Troponin I	0.00 ng/ml	Normal
Creatinine kinase- MB (CK-MB)	0.00 ng/ml	Normal
Creatinine kinase (CK)	24 U/L	Normal

### Imaging findings

**Fig. 2:**
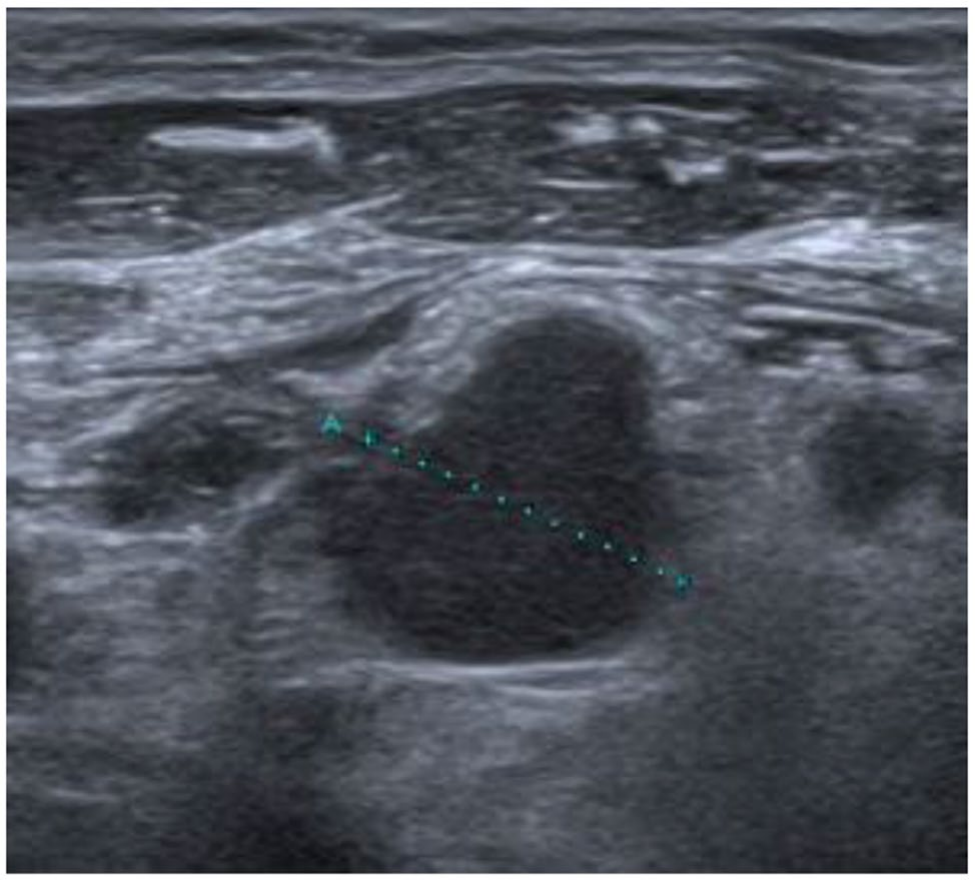
Left neck ultrasound showing left sided lymph node enlargement with loss of fatty hilum

**Fig. 3:**
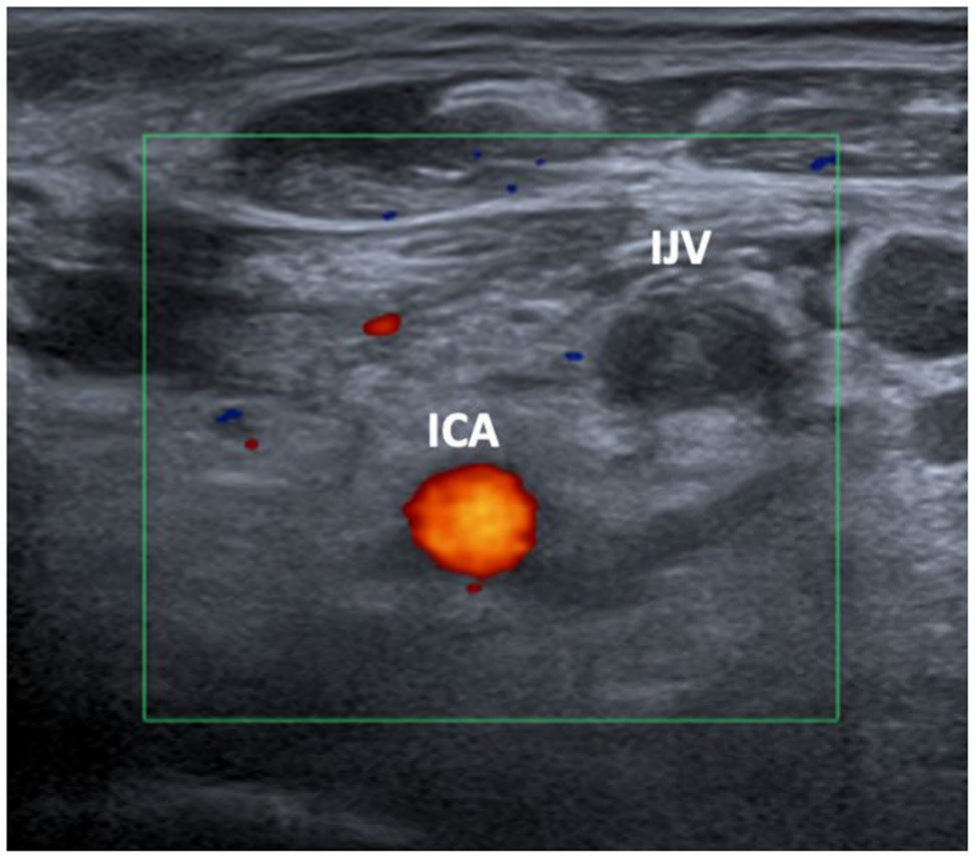
Left neck ultrasound showing a noncompressible left internal jugular vein (IJV) with absent flow in color Doppler images. Normal flow is noted in the internal carotid artery (ICA)

**Fig. 4:**
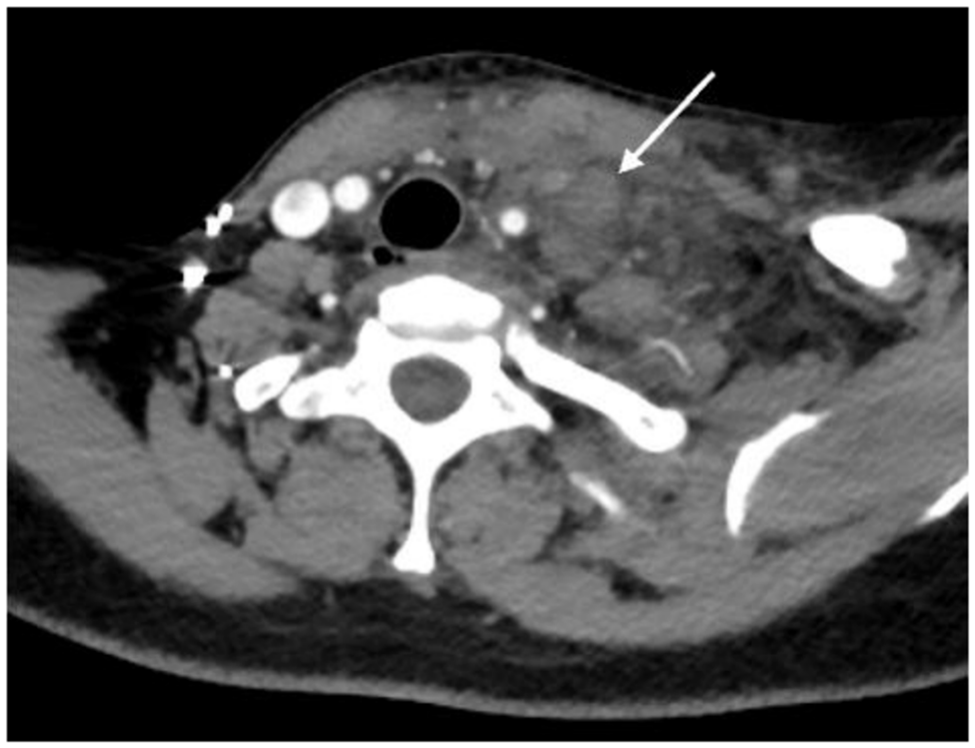
Intravenous (IV) enhanced neck CT showing non-opacification of the left internal jugular, subclavian, and brachiocephalic Veins (white arrow) associated with extensive surrounding fat stranding

Ultrasound of the neck showed multiple bilateral enlarged deep cervical lymph nodes (LN), more prominent on the left side (Fig. 2). The left internal jugular vein (IJV) appeared non-compressible with absent flow on color Doppler images, suggestive of thrombosis (Fig. 3). No abscess or collection was noted. The patient subsequently underwent intravenous (IV) enhanced CT scan of the neck and chest, which showed non-opacification of the left IJV, distal subclavian, and brachiocephalic veins—associated with extensive surrounding fat stranding in the left deep cervical and axillary spaces (Fig. 4). Bilateral enlarged lymph nodes, measuring up to 1.3 cm in short axis. Airway and thoracic structures were otherwise unremarkable. Mild bilateral pleural effusions with subsegmental atelectasis, no septic emboli or abscesses.

### Emergency department management

Paracetamol 1 g intravenous (IV).

Normal saline 500 mL IV.

Piperacillin-tazobactam (Tazocin) 4.5 g IV.

Heparin 4000 IU intravenous bolus initiated due to extensive thrombosis.

ENT and maxillofacial consultation: Airway assessed via fiberoptic scope—patent; no surgical drainage required.

Neurology consultation: No neurological deficit; no intervention needed.

Internal medicine and haematology were consulted for admission and further management.

The patient was admitted to the internal medicine ward for intravenous (IV) antibiotics, anticoagulation, and monitoring.

### Hospital course and follow up

The patient was admitted to the hospital for nine days, during which she received intravenous piperacillin–tazobactam (Tazocin), subcutaneous enoxaparin, and oral lansoprazole. Blood cultures were negative. A thrombophilia workup was performed, which showed a normal prothrombin gene (G20210A) by PCR, heterozygous mutation of factor V Leiden (G1691A) by PCR, and a normal methylenetetrahydrofolate reductase (MTHFR) mutation analysis by PCR. One week after discharge, she was evaluated by an oncologist, and no evidence of malignancy was identified; screening included a mammogram and computed tomography of the chest, abdomen, and pelvis.

## Discussion

Lemierre’s syndrome, first described by André Lemierre in 1936, is a rare form of septic thrombophlebitis of the internal jugular vein that usually follows an oropharyngeal infection, most commonly caused by *Fusobacterium necrophorum*. The incidence of this syndrome markedly declined after the widespread use of antibiotics but has shown a gradual resurgence in recent years, potentially due to increasing antibiotic resistance and changes in empirical prescribing practices.

Atypical presentations without overt signs of sepsis—such as the case presented here—can be diagnostically challenging. Our patient remained afebrile and clinically stable despite extensive venous thrombosis, emphasizing the importance of maintaining a high index of suspicion for Lemierre’s syndrome even in the absence of classical systemic toxicity.

### Diagnosis

Contrast-enhanced computed tomography (CT) of the neck and chest remains the diagnostic modality of choice, as it provides detailed visualization of internal jugular vein thrombosis and associated deep neck infections or abscesses. Ultrasound may serve as an initial screening tool; however, its sensitivity is limited for detecting deep-seated or lower-neck involvement. Blood cultures commonly isolate *Fusobacterium necrophorum*, though they may be negative if antibiotics have been initiated early in the disease course.

### Management

Management requires prompt initiation of prolonged broad-spectrum antimicrobial therapy targeting anaerobic organisms, particularly *Fusobacterium* species. Recommended antibiotic regimens include β-lactam/β-lactamase inhibitor combinations such as piperacillin-tazobactam, ampicillin-sulbactam, or co-amoxiclav.

The role of anticoagulation remains controversial; nonetheless, it is frequently employed when thrombosis is extensive, propagating, or involves major central veins, or in patients at increased risk of thrombophilia as it may have a benefit as was observed in this patient. Emerging evidence suggests that anticoagulation may prevent thrombus progression and enhance venous recanalization without significantly increasing bleeding risk. Direct oral anticoagulants (DOACs), such as rivaroxaban, are gaining attention as practical options for outpatient therapy following stabilization.

### Risk factors for thrombosis

In addition to infection-related thrombosis, other potential prothrombotic risk factors were considered in this patient. She was a smoker and was using a progesterone-only oral contraceptive pill. Smoking is a recognized risk factor for venous thromboembolism, although the risk is generally dose-dependent and higher in heavy smokers. Progesterone-only contraceptives, unlike oestrogen-containing oral contraceptives, are not strongly associated with an increased risk of venous thrombosis. Furthermore, thrombophilia screening in this patient revealed heterozygous factor V Leiden mutation, which may have contributed to the extent of thrombosis. Therefore, it is likely that the thrombosis in this case was multifactorial, with infection, underlying thrombophilia, and minor acquired risk factors contributing to the clinical presentation.

### Multidisciplinary collaboration

Optimal management requires a multidisciplinary approach involving otolaryngology, haematology, and internal medicine teams. Such collaboration is essential for monitoring potential airway compromise, managing sepsis, and identifying underlying prothrombotic risk factors.

### Prognosis

With early recognition and appropriate antimicrobial therapy, the prognosis of Lemierre’s syndrome is generally favourable, and most patients achieve full recovery. Delayed diagnosis or inadequate treatment, however, may lead to severe complications such as pulmonary septic emboli, empyema, or intracranial extension.

## Comparative analysis of case reports with Lemierre’s syndrome

### Patient presentation

Previously reported cases showed significant systemic symptoms. A case reported by Dr. Ali M in Dubai (2025) described a patient with fever, anorexia, headache, abdominal pain, and vomiting, while a case reported by Dr. AlEed in Saudi Arabia (2025) described drowsiness, left mandibular swelling, skin rash, decreased oral intake, and reduced activity. Another case reported by Dr. Aljarrah in Jordan (2018) presented with painful unilateral neck swelling and progressive dysphagia [1, 4, 5]. In contrast, most of these clinical features were absent in our patients, who remained vitally and clinically stable despite extensive thrombosis.

### Investigations

Multiple studies reported elevated white blood cell counts with neutrophilic predominance and elevated C-reactive protein levels, which was similar to our case [1, 4–6]. 

### Imaging

Radiological investigations are essential for confirming the diagnosis, determining the extent of disease, and identifying complications. Contrast-enhanced CT is considered the most appropriate diagnostic modality [7]. 

### Ultrasound of the neck

Dr. Aljarrah (2018) reported a noncompressible, completely thrombosed left internal jugular vein, while another case reported hypoechoic filling within the left internal jugular vein with absent flow and an associated heterogeneous hypoechoic collection. In comparison, our case demonstrated thrombosis of the left internal jugular vein with multiple enlarged deep cervical lymph nodes but no abscess or collection. Other reported cases also demonstrated left internal jugular vein thrombosis, although only one case had an associated collection [5, 6]. 

### Computed tomography (CT)

In 2002, two cases reported in the USA by Dr. Chirinos showed thrombosis of the right internal jugular vein. Another case reported by Dr. Alherabi in 2009 showed bilateral internal jugular vein thrombosis with bilateral pulmonary empyema. The imaging findings in the reported cases are comparable to our case in demonstrating internal jugular vein thrombosis, although our patient had left-sided involvement [2, 8]. 

## Management

### Antibiotics

In ED, there is no time to wait for blood culture; thus, we initiated empirical intravenous (IV) Piperacillin-tazobactam (Tazocin) for broad-spectrum coverage. A similar approach was done by Dr. Aljarrah. While the case in Dubai Ali M, Levofloxacin was added to Piperacillin-tazobactam. On the other hand, Dr. AlEed started with intravenous (IV) ceftriaxone, clindamycin and vancomycin. While a case reported in Ethiopia Dr. Agonafir DB. started with intravenous (IV) Ceftriaxone and Metronidazole [1, 4–7]. Other cases, such as the two reported in the USA in 2002, initiated treatment with clindamycin. In contrast, the case reported Alherabi A. in Saudi Arabia in 2009 started treatment with Penicillin [2, 8]. 

### Anticoagulant

The role of anticoagulation remains controversial; however, it is often used when thrombosis is extensive, propagating, involving major central veins, or in patients at increased risk of thrombophilia, as it may have a benefit as was observed in our patient. We started on Heparin intravenous (IV), which was also employed in other cases [1, 4–7]. 

## Conclusion

This case highlights the importance of maintaining a high index of suspicion for Lemierre’s syndrome in patients presenting with neck pain and swelling following oropharyngeal symptoms, even in the absence of fever or systemic toxicity. Early imaging, prompt initiation of broad-spectrum antimicrobial therapy, and consideration of anticoagulation in selected cases with extensive thrombosis or underlying thrombophilia may be beneficial.

This case also illustrates the importance of a multidisciplinary approach in the management of complex Lemierre’s syndrome cases. Further studies are needed to better define the role of anticoagulation and multidisciplinary management in improving patient outcomes.

### Learning points

Lemierre’s syndrome may present without fever or obvious sepsis; persistent unilateral neck pain following oropharyngeal infection should prompt imaging.

Ultrasound, followed by intravenous (IV) contrast-enhanced CT, remains the preferred diagnostic approach.

Empiric broad-spectrum antibiotics targeting anaerobic bacteria should be initiated promptly.

Anticoagulation is reasonable in cases of extensive central venous thrombosis, or in patients at increased risk of thrombosis as it may be beneficial.

Multidisciplinary collaboration—including Emergency Medicine, Otolaryngologist (ENT), Internal Medicine, and Haematology—is essential for optimal patient management.

## Data Availability

All data generated or analysed during this study are included in this published article.
